# Isolation, Production, and Characterization of Serum Immunoglobulin M (IgM) of Indian Major Carp *Cirrhinus mrigal* (MRIGAL)

**DOI:** 10.1155/2022/2339924

**Published:** 2022-12-20

**Authors:** Prasanna Shama Khandige, K. M. Shankar, P. Suresh Babu, Vandana Sadananda, S. Gowrish

**Affiliations:** ^1^Nitte (Deemed to be University), NGSM Institute of Pharmaceutical Sciences (NGSMIPS), Department of Pharmacology, Mangaluru, Karnataka, India; ^2^Fish Pathology and Biotechnology Laboratory, Department of Aquaculture, College of Fisheries, Karnataka Veterinary Animal and Fisheries Sciences University, Mangaluru, Karnataka, India; ^3^Nitte (Deemed to be University), AB Shetty Memorial Institute of Dental Sciences (ABSMIDS), Department of Conservative Dentistry and Endodontics, Mangaluru, Karnataka, India

## Abstract

A method for the isolation of immunoglobulin M (IgM) in Indian major carp *Cirrhinus mrigal* (mrigal) serum to produce polyclonal antibodies is described in the present study. The purified immunoglobulins (IgM) were isolated from the serum of mrigal (*Cirrhinus mrigal*) by the bovine serum albumin (BSA)-CL affinity column purification method, and the IgM was used to produce a polyclonal rabbit anti-mrigal IgM antiserum. The IgM preparations were employed in the characterization of mrigal serum immunoglobulin. Reduced mrigal IgM on sodium dodecyl sulfate-polyacrylamide gel electrophoresis (SDS-PAGE) was shown to consist of two subunits, compatible with heavy and light chains. A single heavy chain at approximately 90 kDa and variant of light chain 30 kDa were found. The dominant form of nonreduced IgM had a MW of approximately 900 kDa, suggesting a tetrameric structure based on estimated molecular weights, the relative protein content, and the reactivity with anti-mrigal IgM antisera, was obtained. The antisera were characterized as to specificity and reactivity by means of the enzyme linked immuno-sorbent assay (ELISA) and western blotting method. The information on the structure and character of immunoglobulin of fishes is essential in health management. The study described here investigates the possibility of using the serological techniques to assess the reactivity of antibody with the anti-mrigal IgM antisera.

## 1. Introduction

Fish immunoglobulins share various characteristics with mammalian immunoglobulin; however, some differences exist; those partly arise from the process of evolution from common ancestors and partly reflect the effect of fish adaptation to a changed life environment. The fish immune system contains five types of immunoglobulins: IgM [1], IgD [2–4], IgZ [5], IgT [6], and IgH [7]. The major immunoglobulin of fish blood serum is tetrameric IgM-like molecule [7] consisting of eight light and eight heavy chains. The teleost fish IgM shares some structural and functional characteristics with mammalian IgM; hence, we understand the ability of various teleost fishes to produce heterogeneous mixtures of IgM polymers, monomers [2, 8, 9], and halfmer subunits [10].

Wllson et al. [11] had reported severe abdominal dropsy in carps from Meghalaya. Parasitic diseases like myxosporidiosis and mixed infection with saprolegniasis and myxosporidiosis [12] have been accounted in IMC. Numerous occurrences of diseases epidemic of *A. hydrophila* have been reported. [13] Noncommunicable diseases like cataract, tail deformities (lordosis), cholelithiasis, bleeding in eyes, and gas bubble disease have also been reported in IMC. Meticulous knowledge of the molecular structure of the serum IgM heavy chain and the quantification of antibody response in cyprinids are an economically significant component of aquaculture in our country. This will be advantageous for further immunological studies in the field of aqua culture and thereby its application in the field of immunology and inflammation. Majority of the carp's immunoglobulin bear tetrameric structures.

This study presents methods for processing and purification of Indian major carp Cirrhinus mrigal immunoglobulin and its reactivity with anti-mrigal IgM antisera. The antisera were characterized by specificity and reactivity by means of the enzyme linked immuno-sorbent assay (ELISA) and western blotting method.

## 2. Methodology

### 2.1. Isolation of Fish Serum

Five mrigal fishes weighing about 500 g each, maintained in a 25 m [2] cement cistern at the freshwater fish farm of the College of Fisheries, Mangaluru, were injected intraperitoneally (ip) each with 1 mg bovine serum albumin (BSA, Merck, India) dissolved in 250 *μ*l of phosphate buffered saline (PBS, pH 7.4) emulsified with an equal volume of Freund's complete adjuvant (FCA). Similar doses of BSA emulsified with an equal volume of Freund's incomplete adjuvant (FIA) were given 15 and 22 days later. Two mrigal fishes injected with PBS (pH 7.4) alone were maintained as control for the collection of unimmunized serum. On the 30th day, fishes were anaesthetized with 10 ppm benzocaine for 3 min followed by the collection of blood by puncturing the caudal vein, which was allowed to clot for 1 h at room temperature and stored overnight at 4°C. Serum was collected after centrifuging at 6000 rpm for 10 min and pooled and stored at −20°C for further use [2].

### 2.2. Purification of Serum

Purification of serum was carried out by affinity column chromatography according to Gopalakrishnan et al. [13], using a 5 ml BSA-CL agarose column (Bangalore Genei, India). The column was equilibrated by passing 5 column volume of PBS (pH 7.2). Two milliliters of serum from immunized fish, mixed with an equal volume of PBS (pH 7.2), was filtered through a 0.45 *μ*m syringe filter (MDI, India) and loaded onto the column. The serum was allowed to interact with the BSA for 45 min. Unbound serum protein was washed out with 10 column volumes of elution buffer (0.1 M citric acid buffer, pH 3.0). Approximately, 10 fractions of 0.55 ml each were collected and neutralized with 0.45 ml of 0.5 M Tris buffer (pH11). Absorbance at 280 nm of each fraction was measured spectrophotometrically. The eluted samples were pooled and subjected to dialysis at 4°C against PBS using a low molecular weight (12 kDa cut off) dialysis membrane (Sigma, USA). The dialysis membrane was shifted to a tray containing sucrose to concentrate it to 2 ml, and the concentrated IgM was stored at −20°C for further use.

### 2.3. Characterization of Purified IgM

Purified IgM was characterized by nonreducing SDS-PAGE, briefly 10 *μ*l of the 4x native PAGE sample buffer (10% SDS, 0.5 M Tris-HCl, pH 6.8), glycerol 0.8 ml, and 0.01% bromophenol blue (W/V) was added to 30 *μ*l of the purified IgM. The purified IgM along with a molecular weight marker (Sigma, USA) and native human IgM (Sigma, USA) were loaded onto the gel containing 3% acrylamide and 0.5% agarose and subjected to electrophoresis at 25 mA till the dye front reached the bottom of the gel which was strained with coomassie blue (10% acetic acid, 40% methanol, 0.1% coomassie blue) for 8 h and destained with destaining solution (10% acetic acid, 40% methanol), and the molecular weight of the IgM was estimated. Further characterization of the IgM was carried out by 10% SDS-PAGE according to Laemmli [14]. About 10 *μ*l of the 4X sample buffer (10% SDS, 2 –*β*-mercapto ethanol. 0.4 ml, 0.5 M tris-HCl, pH 6.8), glycerol 0.8 ml, and 0.01% bromophenol blue (W/V) was added to 30 *μ*l of the purified IgM and boiled at 100°C for 5 mins. For comparison, crude serum from an immunized fish was prepared in a similar way. The samples along with molecular weight markers (Sigma, USA) were loaded onto 4.5% stacking gel and subjected to electrophoresis at 30 mA in 10% separating gel till the dye front reached the bottom of the gel. The gel was stained with coomassie blue, and the molecular weight of heavy and light chain Ig were determined [2].

### 2.4. Production of Polyclonal Antibody

A polyclonal antibody-based ELISA method was adapted for the detection of mrigal antibodies. Ten-week old Swiss albino mice were immunized (ip) with purified mrigal IgM (50 *μ*g in 250 *μ*l PBS) mixed with 1 : 1 with FCA. A similar second dose (ip) was given with the purified IgM in FIA (1 : 1) on the 15th day. On the 27th day, a booster dose of 10 *μ*g purified IgM in 0.05 ml PBS was given intravenously. Blood was withdrawn on the 30th day from the tail vein using a hypodermic syringe and collected blood was allowed to clot for 1 h at room temperature. Later, the serum was obtained by centrifuging the blood clot at 6000 rpm for a period of 10 mins, which was used as a polyclonal antibody.

### 2.5. Enzyme Linked Immunosorbent Assay (ELISA)

Indirect ELISA was performed to analyze the BSA specific antibody activity in immunoaffinity chromatographic fractions pf pooled mrigal sera as described by Towbin et al. [15]. Microtiter plates (Nunc, Denmark) were coated overnight with 1% BSA in carbonate buffer (15 mM Na_2_CO_3_, 35 mM NaHCO_3_, pH 9.6) followed by (1) blocking with 5% skimmed milk powder in PBS with 0.1% Tween 20 (PBS/Tween), (2) incubation with doubling dilutions (1 : 100 to 1 : 12400) of purified Ig, immunized sera and unimmunized sera in PBS/Tween in the two sets, (3) incubation with polyclonal mice anti-mrigal IgM antiserum (1 : 1000 in PBS/Tween), (4) incubation with horseradish peroxidase conjugated rabbit anti-mouse Ig antiserum (Bangalore Genei, India) (1 : 1000 in PBS/Tween), and (5) incubation for 10 mins with TMB H_2_O_2_ (Bangalore Genei, India) (1 : 20 in PBS/Tween). The reaction was stopped by addition of 1M H_2_SO_4_ and the colour development was observed at 490 nm (OD490) which was measured with ELISA reader (Bio-Tek, USA). All incubations were for 1h at room temperature. All the steps between (1) and (5) were separated by three washes with PBS/Tween.

### 2.6. Western Blotting

Purified mrigal IgM was solubilized in the sample buffer under reduced conditions. After separation in 10% polyacrylamide gel [16], the proteins were transferred onto the nitrocellulose membrane [14]. After drying, the nitrocellulose strips were incubated with polyclonal antibodies diluted by 1 : 10000. The reaction was detected using rabbit anti-mouse IgG antibodies labeled with horse radish peroxidase.

### 2.7. Polyclonal Antibody (Pab) Based Immunodot Assay for the Study Reactivity of Mrigal IgM

An immunodot assay was carried out by dotting BSA onto nitrocellulose (N.C.) papers cut into 02 cm^2^ size. Purified IgM was dotted on the paper as positive control and *Aeromonas hydrophila* and vibrio sp bacteria were dotted as a negative control, and three such dotted membranes were prepared separately to detect antibodies in the affinity purified IgM sample, crude immunized, and unimmunized sera. Free sites on the nitrocellulose membrane were blocked with 05% skimmed milk powder in PBS for 02 hours and washed 03 times with PBS (pH 7.4), and one of the membranes was treated over night with 01 ml of purified IgM and at a concentration of 0.1 mg/ml, and the other with crude immunized fish serum diluted to 10 times with PBS (01 ml). The third membrane was treated overnight with unimmunized fish serum diluted to 10 times with PBS (01 ml). After washing with PBS Tween 20, rabbit anti-mouse IgG horse radish peroxidase (Bangalore genie, India) in 3% BSA in PBS (1 ml, 1 : 1000 dilution) was added and incubated for 90 minutes. After washing with PBS Tween 20 three times, 1 ml of substrate (0.3 mg of 4 chloro-1-naphthol in 10 *μ*l ethanol, 01 *μ*l of 30% H_2_O_2_, and 01 ml of Tris buffer (pH 7.6)) was added, and clear purple dot development on the nitrocellulose paper was considered as positive reaction.

## 3. Results

**Figure 1:**
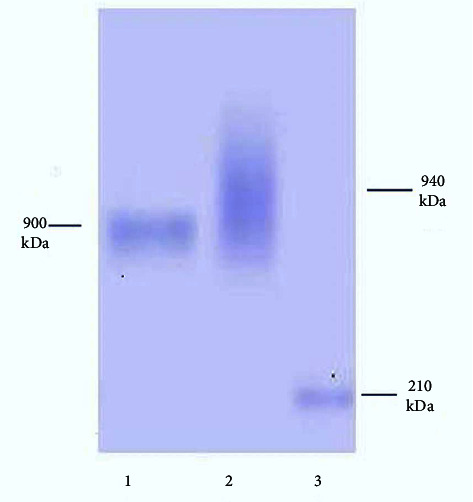
SDS-PAGE (native) under non reduced condition (3%). Lane 1 purified IgM. Lane 2 Human IgM. Lane 3 molecular marker.

**Figure 2:**
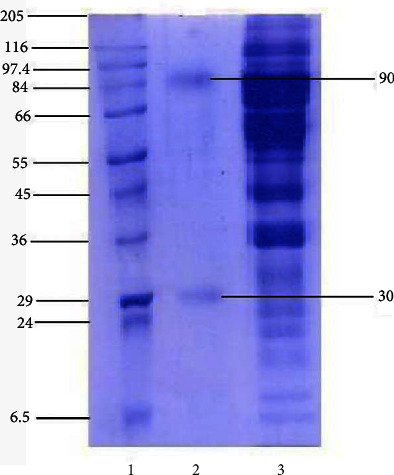
SDS PAGE under reduced condition. Lane 1: molecular marker. Lane 2: purified IgM. Lane 3: crude mrigal serum.

**Figure 3:**
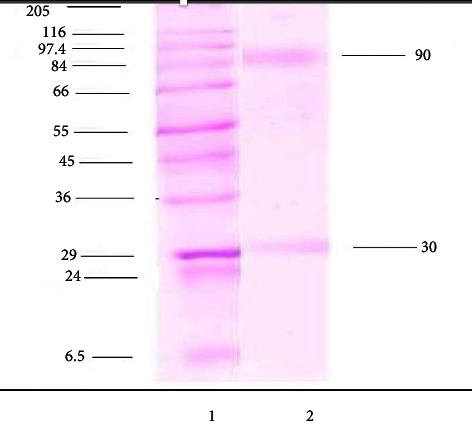
Western blot analysis. Lane 1: molecular marker. Lane 2: affinity purified mrigal IgM.

**Figure 4:**
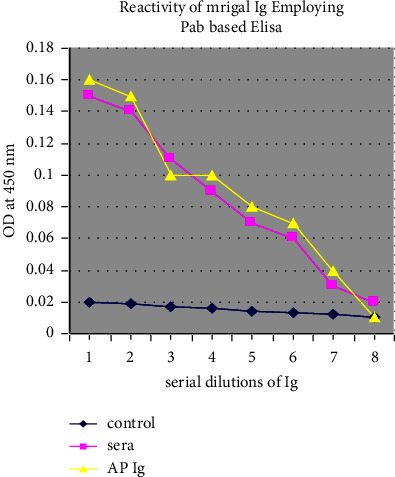
Reactivity study by PAB based ELISA.

**Figure 5:**
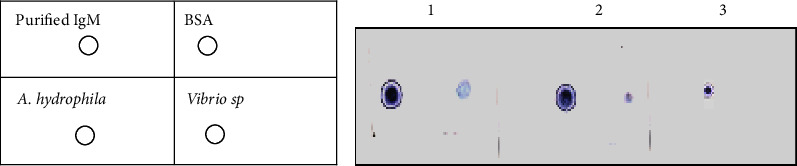
Immunodot assay. 1. Immunoglobulin IgM. 2. Sera. 3. Unimmunize.

Fish serum was obtained after immunization with bovine serum albumin (BSA), the sera obtained was purified by affinity column chromatography method eluted fractions and characterized by poly acrylamide gel electrophoresis (PAGE) under reducing and nonreducing conditions (Figures 1 and 2). Polyacrylamide gel electrophoresis (PAGE) under reduced condition revealed that the immunoglobulin contained heavy and light chains with molecular weights of 90 and 30 kDa, respectively, and under nonreducing conditions in a 3% gel revealed a single protein having a molecular weight of 900 kDa. The method was further employed for production of polyclonal antibodies against anti-mrigal serum in the mouse, and thus, polyclonal antibodies against mrigal anti serum was obtained. The sensitivity and the titer were determined using ELISA. The antibody titer was found to be 1 : 12400. The antibody sensitivity is significant in purified mrigal IgM while compared with crude mrigal antiserum, unimmunized negative control serum did not react. In dot immuno blot assay also, mrigal polyclonal antibodies reacted significantly with the pure mrigal IgM while compared to crude mrigal antiserum, and unimmunized negative control sera did not react. Similarly, in western blot analysis of mrigal IgM, two distinctive bands showing heavy and light chain was observed, the molecular weight of heavy chain was 90 kDa and light chain was 30 kD, which reacted significantly towards pure mrigal IgM and crude mrigal antiserum, and unimmunized negative control sera had not reacted (Figures 3–5).

## 4. Discussion

In the present study, we tried to purify the immunoglobulin of mrigal by a single step affinity purification by the BSA-linked column, which was suitable to get adequate amount of immunoglobulin (Ig); moreover, the same column was found to be useful to purify more than once without reduction in the binding capacity of the antibody adapting established immunization protocol and the purification technique, and an average yield of immunoglobulin (Ig) was 1.25 mg/ml of serum. Ig levels in different fishes after immunization vary within the range of 0.25–23.5 mg/ml [11].

The immuno dot test clearly showed specific reaction of the antibodies in the affinity-purified IgM and crude antisera with BSA. In the NC membrane treated with affinity-purified IgM (Figure 5) and the membrane treated with crude immunized serum, the BSA spots were clearly stained indicating the specific reactivity of immunized crude antiserum and purified IgM. The membrane treated with unimmunized mrigal serum did not develop purple blue dot with BSA. The positive control with dot of purified IgM also developed purple colour in all sets of assays. The negative controls (*A. hydrophila* and *Vibrio* sp. Bacterins) did not show any colour reaction.

In the present study, only one form of IgM-like molecule was eluted in the affinity purification. In the earlier studies on *Cirrhinus mrigal* (mrigal), *Catla catla* (Catla), and *Lobeo rohita* (rohu) with nonreducing SDS-PAGE, different populations of Ig molecules such as tetrameric and dimeric ano-monomeric forms have been reported, but we have observed a single band of protein in nonreducing SDS-PAGE indicating that protein is pure and was eluted in its native form. The molecular weight of *C. mrigal* IgM was 900 kDa in nonreducing SDS-PAGE, which is similar to the molecular weight of IgM of the fishes such as Turbot (835 kDa African catfish 840 kDa Cod 851 kDa).

SDS-PAGE analysis revealed two clear bands of polypeptides having molecular weights of 90 and 30 kDa which are considered as the heavy and light chains of the purified IgM molecule, respectively. The immunized crude serum also shows the corresponding heavy and light chain bands. The molecular weights of heavy and light chains can be compared with those from other species of fishes like Tilapia (90 and 30 kDa), Cod (811 and 27.5 kDa), Trematomus bernacchii (83.5 and 27.5 kDa), Turbot (78 and 27 kDa), and common carp (70 and 25 kDa) [16].

The present paper describes the attempts made and strategy employed to obtain specific polyclonal antibody against mrigal antiserum to develop a strategy to obtain a specific polyclonal antibody against mrigal antiserum and to develop a sensitive, rapid, and specific ELISA to employ in routine screening in disease management. In the present study, BSA antigens were selected to raise the antibodies in mrigal because of the accessibility of affinity columns for antibody purification. BSA has been used as an immunogen for elucidating antibody production in different fishes by many investigators [16–18].

Eluted fractions of immunoglobulin were analyzed afterwards by the antiserum which was found extremely immunogenic as assessed by the titration of immune sera. The ELISA developed was shown to be very sensitive with titer of 1 : 12400. The antibody reacted more strongly with pure mrigal IgM than with crude mrigal sera as ELISA, immmunodot, and western blot has confirmed the results [13, 19].

## Data Availability

The data used to support the study are included in the paper.
